# Disease Burden of RSV Infection in Adult Patients in Comparison to Influenza Virus Infection

**DOI:** 10.1002/jmv.70373

**Published:** 2025-04-30

**Authors:** Georgii Trifonov, Erik Büscher, David Fistera, Clemens Kill, Joachim Risse, Christian Taube, Daniel Todt, Ulf Dittmer, Carina Elsner

**Affiliations:** ^1^ Institute for Virology University Hospital Essen, University of Duisburg‐Essen Essen Germany; ^2^ Department of Pulmonary Medicine University Medicine Essen‐Ruhrlandklinik Essen Germany; ^3^ Center of Emergency Medicine University Hospital Essen Essen Germany; ^4^ Department of Translational and Computational Infection Research Ruhr University Bochum Bochum Germany; ^5^ European Virus Bioinformatics Center (EVBC) Jena Germany

**Keywords:** clinical study, influenza virus, respiratory syncytial virus, RSV, vaccine

## Abstract

Respiratory Syncytial Virus (RSV) is well known for its impact on children, but its burden in adults remains underexplored, partly due to limited PCR testing before the COVID‐19 pandemic. In this study, the medical burden of RSV infections in adults was retrospectively investigated using 6‐year longitudinal data from a university hospital in North Rhine‐Westphalia, Germany. Outcomes of 380 PCR‐confirmed RSV cases were compared with 1088 influenza A/B cases from 2018 to 2023, stratified by age groups ( < 60 and ≥ 60 years). Among RSV cases, 59.7% required hospitalization, of which 22.9% needed oxygen supply. In the whole group hospitalization rates were comparable between RSV and influenza cases, but oxygen supply was more frequent in influenza infections. However, in patients aged ≥ 60 years, no significant differences were observed in hospitalization, oxygen supply, or fatal outcomes between RSV and influenza, indicating a comparable disease burden for both viruses in this group. These findings highlight the significant clinical impact of RSV in adults, particularly those aged ≥ 60 years, paralleling that of influenza. Given influenza's established pathogenic reputation, this underscores the importance of targeted vaccination strategies against RSV, especially for high‐risk age groups.

## 1 Introduction

Respiratory virus infections are a seasonal global challenge, affecting individuals across all age groups. Respiratory Syncytial Virus (RSV), a member of the *Pneumoviridae* family, is a widespread pathogen known for its substantial impact on vulnerable populations, particularly young children [1]. However, there is growing evidence that RSV also induces severe infections in the elderly. Consequently, RSV is one of the most common causes of acute respiratory infections, significantly contributing to morbidity and mortality among children, the elderly, and immunocompromised individuals [2, 3]. In adults, RSV infections can vary in severity, ranging from asymptomatic infections or mild respiratory symptoms to severe lower respiratory tract infections (LRTIs) [3, 4]. For older individuals and those with underlying medical conditions, it is known that RSV infection can necessitate hospitalization even with fatal outcome [5].

In 2019, an estimated 5.2 million cases of RSV‐related acute respiratory illness were reported among older adults in industrialized countries, with approximately 9% of these cases leading to hospitalization [6]. Interestingly, the regulatory measures implemented during the COVID‐19 pandemic also had a sustained impact on other respiratory pathogens, such as RSV and influenza viruses. During the winter seasons of 2020/2021, both pathogens were almost undetectable, and in the following season of 2021/2022, their detection remained minimal [7]. However, due to the anticipated waning of immunity, an early and intense wave of infections was observed for both influenza virus and RSV during the 2022/2023 season. While extensive research has been conducted on the impact of RSV in pediatric populations [8, 9], the RSV burden of disease in the elderly has received comparatively less attention. RSV infections in the adult population present a growing global health burden, particularly in high‐income countries where aging populations experience increased hospitalizations and healthcare costs. In 2019, the global mortality rates from RSV in individuals over 70 years surpassed those in children under five, highlighting the virus's severe impact on the elderly [10]. Hospitalizations due to RSV often resulted in increased healthcare costs, especially among patients with comorbidities. Implementing preventive measures, such as vaccination, could alleviate healthcare costs and improve clinical outcomes for this vulnerable population [11]. However, recent studies underscore the importance of understanding RSV infection dynamics in this population, given its likely significant implications for health resource utilization, morbidity, and mortality [12, 13].

Recent advancements in RSV vaccine development for adults have led to the approval of several vaccines in late 2023 and 2024. Pfizer's bivalent vaccine, Abrysvo, contains two recombinant RSV fusion surface glycoproteins [14], while GSK's monovalent vaccine, Arexvy, includes a recombinant RSV F protein antigen stabilized in its prefusion structure [15, 16]. The third approved vaccine, mRESVIA by Moderna, uses an RNA‐based platform with an mRNA sequence that encodes for a stabilized prefusion F glycoprotein [17]. All three vaccines are authorized for individuals aged ≥ 60 years. However, further research is essential to identify the age groups that would most benefit from vaccination. Such findings will play a critical role in shaping national vaccination guidelines and health insurance coverage within RSV immunization programs. The winter season 2024/2025 may provide preliminary real‐world efficacy data, yet it remains vital to thoroughly assess the burden of RSV disease across adult age groups and among those with comorbid conditions.

Due to the limited number of studies most European countries, including Germany, have so far recommended vaccination from the age of 75 or for people between 60 and 74 with pre‐existing conditions. However, there is evidence that RSV infection leads to a significant disease burden, hospitalization, and even mortality, comparable to the outcomes of influenza virus infections, particularly in individuals aged ≥ 60 years and regardless of comorbidities [6, 12, 13, 18]. For this reason, some European countries, such as Austria, have a general RSV vaccination recommendation for people aged 60 and older [19].

This retrospective observational study examines the disease burden associated with RSV infection in adults aged 18 years and older before the introduction of RSV vaccines. Understanding the pre‐vaccine burden of RSV disease is critical for the optimal use of the currently approved vaccines for adults. Our study provides a comparative assessment of RSV and Influenza A/B (IAV/IBV) infections, with a particular focus on adults aged ≥ 60 years—a population at risk for severe infectious disease outcomes. By highlighting the clinical manifestations and complications of RSV infection in this demographic, our findings help to fill key gaps in the literature and provide essential evidence to guide vaccination strategies and resource allocation in healthcare systems.

## 2 Methods

### 2.1 Study Population and Data Collection

We conducted a retrospective observational study utilizing passively collected data from virology databases and medical records at a single tertiary care hospital. The hospital's catchment area includes a densely populated metropolitan area in Western Germany, characterized by a diverse demographic profile, including various ages groups, socioeconomic backgrounds and a large number of patients with pre‐existing diseases and conditions, due to the specialist focus of the hospital.

Data was collected retrospectively from 2018 until June 2023. Our routine diagnostic virology database was screened for patients at the age of ≥ 18 years with at least one positive RSV and/or Influenza virus A or B RT‐PCR test. Overall factors known to influence disease progression and mortality of respiratory infections, such as age, gender, outcome and oxygen supply status (including both invasive and noninvasive oxygen supply) were extracted from patient records by Medical Controlling at University Hospital Essen.

For both cohorts additional information, such as immune status and comorbidities (diabetes, chronic cardiac insufficiency, chronic obstructive pulmonary disease [COPD], hypertension, chronic heart disease [CHD]) was collected from patient records.

### 2.2 Testing for Respiratory Viruses

Before the COVID‐19 pandemic respiratory viruses were diagnosed when clinically indicated due to respiratory symptoms. However, for RSV in adults, tests were in many cases only performed when patients had pre‐existing lung diseases and respiratory symptoms. During and after the pandemic, all patients presenting to the hospital's ICU underwent routine screening for respiratory pathogens during the winter season. Outside of this period, testing was performed based on clinical indications. Nasopharyngeal swabs were collected and reverse transcription polymerase chain reactions (RT‐PCR) for RSV, Influenza A and Influenza B viruses were performed with CE‐IVD‐labeled diagnostic tests. Several different diagnostic tests were used during the retrospective analysis period (Table 1). All assays used were CE‐IVD‐labeled tests validated for our accredited diagnostic laboratory, ensuring consistent quality, sensitivity, and specificity throughout the study period. Inter‐assay comparisons were performed according to our quality management system.

**Table 1:** Overview of all diagnostic tests used in 2018 until June 2023 for the diagnosis of RSV or Influenza A/B virus.

Test name	Company	Nucleic acid extraction	Company	Used since	Used until
FTD Respiratory pathogens 21	Fast‐track diagnostics, luxembourg/siemens healthineers, Germany	MagNA Pure 96 DNA and viral Small volume kit	F. Hoffmann‐La Roche Ltd., Swiss	06/2013	
FTD HRSV	Fast‐track diagnostics, Luxembourg	MagNA Pure 96 DNA and viral Small volume kit	F. Hoffmann‐La Roche Ltd., Swiss	01/2014	11/2020
FTD Flu/HRSV	Siemens healthineers, Germany	MagNA Pure 96 DNA and viral Small volume kit	F. Hoffmann‐La Roche Ltd., Swiss	11/2020	01/2024
Xpert Xpress Flu/RSV	Cepheid Inc., USA	GeneExpert	Cepheid Inc., USA	12/2018	03/2023
Xpert Xpress CoV2/Flu/RSV	Cepheid Inc., USA	GeneExpert	Cepheid Inc., USA	01/2021	02/2022
Xpert Xpress CoV2/Flu/RSV plus	Cepheid Inc., USA	GeneExpert	Cepheid Inc., USA	02/2022	
Alinity m RESP‐4‐PLEX assay	Abbott, USA	Alinity m	Abbott, USA	08/2023	

### 2.3 Calculation of Infection‐Associated Mortality Rate

The infection‐associated mortality rate was calculated for both RSV‐ and IAV/IBV‐infected cohorts using the following formular:

(1) $$\text{Infection associated mortality rate}\;[\%]=\frac{\text{Deaths of hospitalized patients due to infection}}{\text{Total number of infected hospitalized patients}}\times 100$$

### 2.4 Statistics

Descriptive statistics were calculated to summarize the data, including percentages for categorical variables and measures of central tendency, such as mean and median, for continuous variables. Fisher's exact test was employed to analyze associations between categorical variables, ensuring robust results even with small sample sizes using GraphPad Prism version 10.4.0 for Windows (GraphPad Software, Boston, Massachusetts USA, www.graphpad.com). Multiple logistic regression analysis was conducted to assess the relationship between the dependent variable and one or more independent variables, adjusting for potential confounders using DATAtab: Online Statistics Calculator (DATAtab e.U. Graz, Austria, https://datatab.de). *P* values < 0.05 were considered statistically significant.

### 2.5 Ethics

The present study was reviewed and approved by the Ethics Committee of the Faculty of Medicine of the University of Duisburg‐Essen (22‐10779‐BO).

## 3 Results

### 3.1 Main Characteristics of the Patient Cohort at the University Medicine Essen

**Figure 1:**
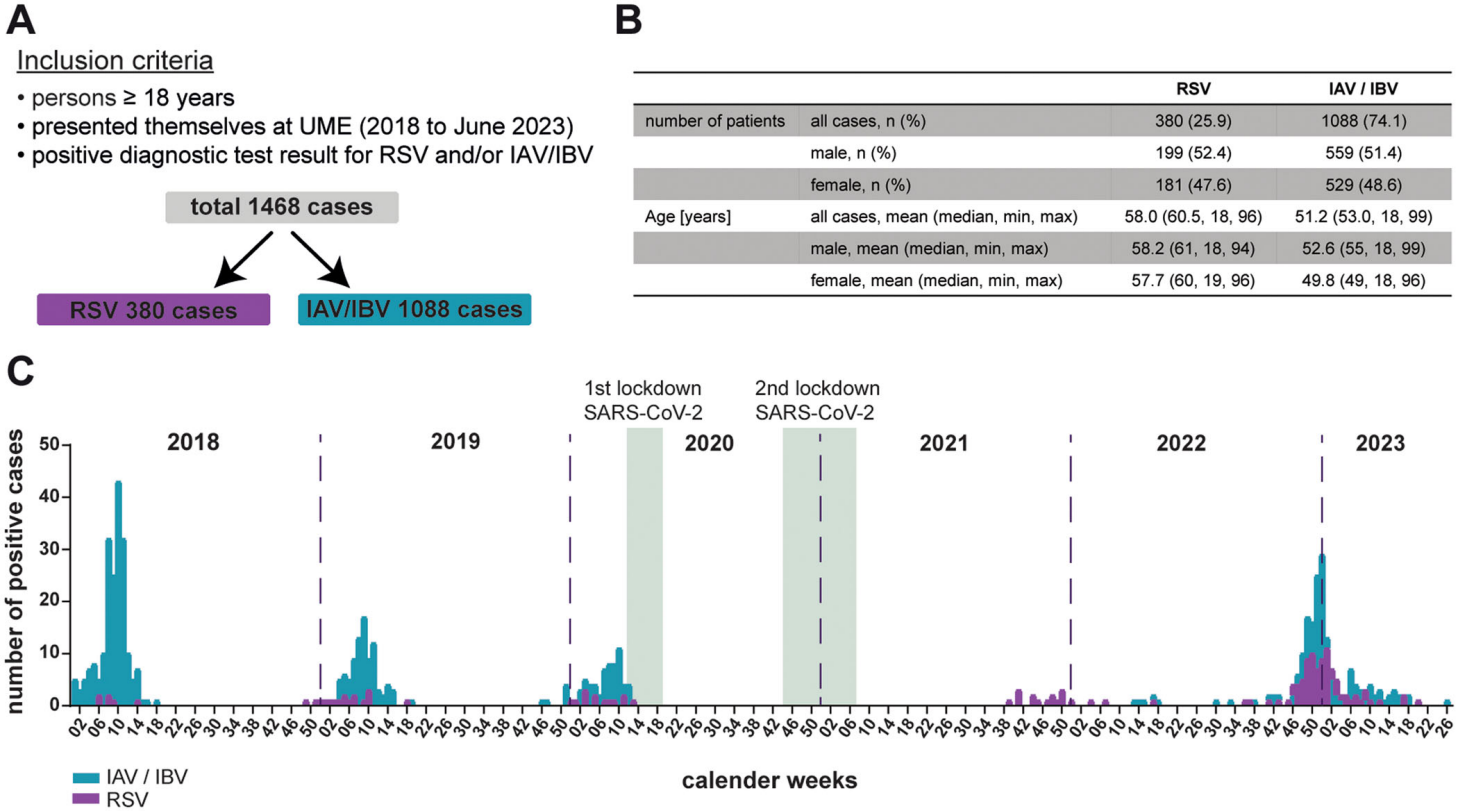
Characteristics of the University Medicine Essen (UME) Respiratory Syncytial virus (RSV) or Influenza A/B virus (IAV/IBV) positive cohort. (A) Summary of inclusion criteria for the retrospective analysis. (B) Main characteristics of the cohort, depicted are number of patients and distribution of age, subdivided in male and female (numbers (*n*) and percentages), as well for age (median, minimal (min) and maximal (max) values in years). (C) Course of RSV (purple) and IAV/IBV (turquoise) infection waves over the time period 2018 until mid‐2024 in calendar weeks. Depicted are the number of positive tested cases. Both, first and second lockdown caused by SARS‐CoV‐2 are highlighted (greenish boxes).

We retrospectively selected a cohort of RSV or influenza virus positive patients at the University Medical Center Essen (UME), a tertiary care hospital, to investigate the disease burden of RSV‐infected individuals. Our study included individuals aged 18 years or older who presented themselves to the UME between 2018 and June 2023 and received a positive diagnostic PCR test for either respiratory syncytial virus (RSV) or Influenza A or B virus (IAV/IBV) (Figure 1A). In total, 1468 cases were identified, comprising 380 RSV‐positive cases (25.9%) and 1088 IAV/IBV‐positive cases (74.1%). The much larger number of IAV/IBV‐positive cases likely reflects differences in viral distribution within the population, but also an inequality in test frequencies before the pandemic started. Among the infected patients, there were also 17 RSV‐infected patients simultaneously infected with SARS‐CoV‐2 and one RSV‐infected patient had an Influenza A virus co‐infection. Due to the very small number of coinfected patients, they were not excluded from the analysis. Both the RSV and IAV/IBV‐positive groups had nearly equal gender distribution, with 52.4% of the RSV cases being male and 47.6% female, and 51.4% of the IAV/IBV cases being male and 48.6% female. The average age of the RSV group was 58 years (median 60.5 years), while the IAV/IBV group was slightly younger, with a mean age of 51.2 years (median 53 years) (Figure 1B, Supporting Information S1: Figure 1). Overall, both cohorts differed in their age distribution, with a higher number of younger patients (18–50 years) infected with IAV/IVB (Supporting Information S1: Figure 1).

During the COVID‐19 pandemic, greater emphasis was placed on routine testing for respiratory viruses in hospitals, including RSV and IAV/IBV testing in a point‐of‐care PCR detection system. This increased the number of diagnostic tests and was reflected in the number of positive cases identified in the post‐pandemic season between 2022 and 2023. It is well‐established that both RSV and IAV/IBV infections follow a seasonal pattern, which we also observed in our center (Figure 1C). Interestingly, a shift for the RSV and influenza virus season was observed after the second COVID‐19 pandemic lockdown in Germany, with RSV infections beginning much earlier in the 2021/2022 season, starting in calendar week 39 rather in 51/52. This trend continued for both RSV and IAV/IBV during the 2022/2023 season (Figure 1C). A notable increase in IAV/IBV cases was observed in 2018 compared to the pre‐pandemic seasons 2019 and 2020, with a particularly severe IBV season in 2018.

### 3.2 The Disease Burden and Outcome of Hospitalized RSV‐Infected Patients Is Comparable to That of IAV/IBV‐Infected Patients

**Figure 2:**
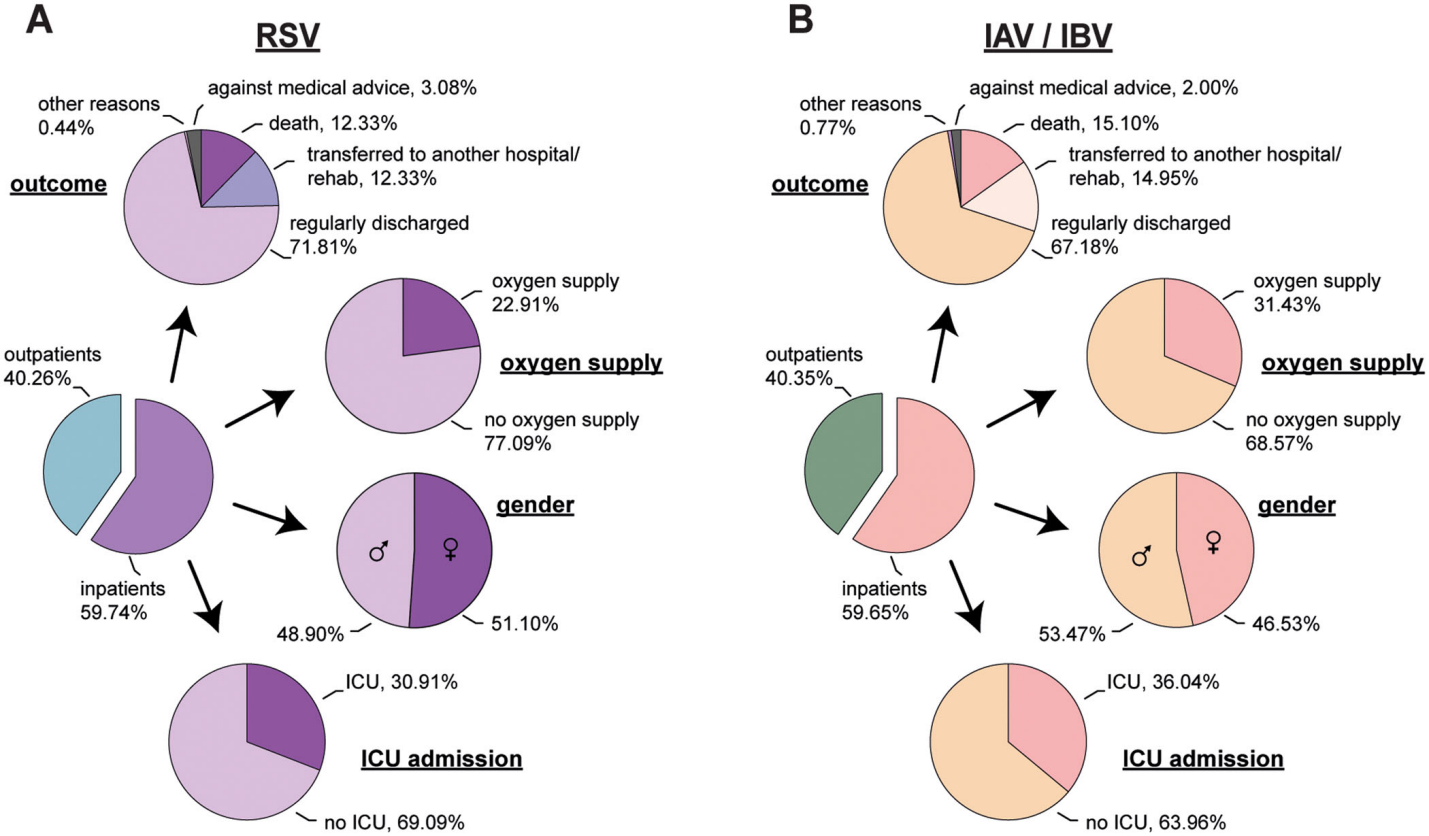
Patient characteristics. Characteristics of patients tested positive by PCR for RSV (A) or IAV/IBV (B). Shown are the proportion of hospitalized patients, the outcome, oxygen supply status, and gender and intensive care unit (ICU) admission rates in percentage.

To assess the disease burden of RSV infection in our cohort, we compared the patients tested positive by PCR to those infected with IAV/IBV infection during the same time period at the UME. Overall, 59.7% (*n* = 227) of RSV‐infected patients were hospitalized, with 22.91% (*n* = 52) requiring oxygen supply (including both noninvasive and invasive oxygen support) (Figure 2A and Supporting Information S1: Table 1). The fraction of hospitalized patients was absolutely comparable to the IAV/IBV‐infected cohort, where 59.65% (*n* = 649) of patients were hospitalized. The frequency of ventilated patients was higher after IAV/IBV infection, with 31.43% (*n* = 204) requiring oxygen supply (Figure 2B and Supporting Information S1: Table 1). Overall, the requirement for oxygen supply was significantly lower after RSV infection compared to IAV/IVB (*p* value 0.017, Fisher's exact test). In addition, 30.91% of the RSV‐infected patients were admitted to the intensive care unit (ICU), a rate similar to that of influenza‐infected patients, of whom 36.04% required intensive care treatment (Figure 2A,B). Overall, ICU‐admitted patients with IAV/IBV infection were more likely to require invasive ventilation (IV) compared to RSV infection (*p* = 0.0214, Fisher's exact test; Supporting Information S1: Table 1). The majority of the patients could be regularly discharged from the hospital, with discharge rates of 71.81% for RSV‐infected and 67.18% for IAV/IBV‐infected cases, respectively. However, the mortality rates (formula 1) for both infections were notable, with 12.33% for RSV‐infected and slightly higher 15.10% for IAV/IBV‐infected patients. Some patients of both groups (RSV: 12.33% and IAV/IBV: 14.95%) were transferred to other hospitals and could therefore not be followed up anymore. It is possible that also some of these patients died due to severe disease. Interestingly, there were no obvious gender differences in either population, although there was a slight trend towards more hospitalized women in the RSV cohort (51.1% and IAV/IBV 46.53%) and, in contrast, slightly more men in the IAV/IBV group (53.47% and RSV 48.90%). If this was influenced by lower overall patient numbers in the RSV group remains unclear. Overall, there were no significant differences in hospitalization rates or clinical outcomes between RSV and IAV/IBV‐infected patients over 18 years of age, except for a higher rate of oxygen supply use in the IAV/IBV group.

### 3.3 RSV Infection Is Particularly Dangerous for Patients Over 60 Years of Age

Age is a well‐established risk factor for a poor outcome in infections with respiratory viruses. While there is evidence that this is also the case in RSV infection of adults, there is much less data available as for example for influenza or Coronaviruses. Accordingly, there are recommendations in place in many countries for a vaccination against COVID‐19 and influenza virus starting at the age of 60 years.

**Figure 3:**
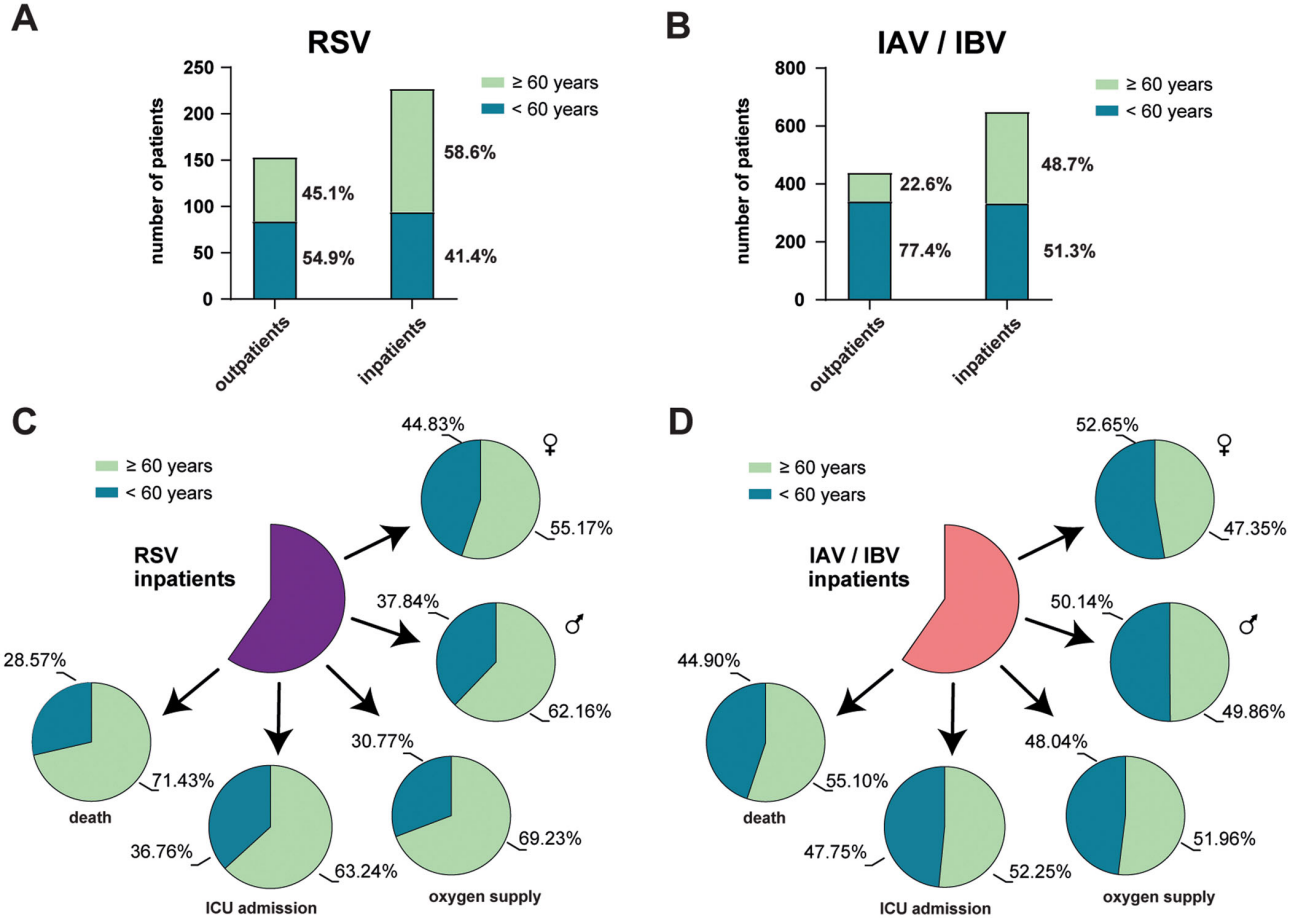
Age distribution in the cohort within two groups, younger than 60 years (dark green) and 60 years and older (light green). (A and B) Percentage of in‐ and outpatients in both age groups are shown for RSV‐infected patients (A) and IAV/IBV infected patients (B). (C and D) Proportion of males and females, as well as number of patients that needed oxygen supply, were admitted to ICU or died are presented for both age groups in percentages. RSV‐infected patients in (C) and IAV/IBV‐infected cohort in (D).

To this end, we re‐analyzed our cohorts based on the two age groups < 60 and ≥ 60 years according to their hospitalization, oxygen supply status, and clinical outcomes, with a comparison between RSV‐ and IAV/IBV‐infected patients. Notably, in the RSV‐infected cohort, almost 60% of all inpatients were ≥ 60 years old (Figure 3A and Supporting Information S1: Table 2). In contrast, in the IAV/IBV‐infected cohort we found a more balanced distribution between the two age groups, with slightly more patients under 60 years (< 60 years: 51.31%; ≥ 60 years: 48.69%) (Figure 3B and Supporting Information S1: Table 2). Thus, the probability of being hospitalized at the age of ≥ 60 years due to an RSV infection is significantly higher compared to an IAV/IVB infection (*p*‐value 0.01, Fisher's exact test). Among the outpatients, who were discharged from the hospital after their initial test and an acute treatment, there was a notably higher proportion of IAV/IBV‐infected patients under 60 years compared to those over 60 years (< 60 years: 77.45%; ≥ 60 years: 22.55%) (Figure 3B). In the RSV cohort, both age groups were almost equally represented among outpatients (< 60 years: 54.90%; ≥ 60 years: 45.10%; Figure 3A).

**Table 2:** Immune status and comorbidities of RSV‐infected patients. Summarized are the number of immunosuppressed patients and frequency of comorbidities in all RSV‐infected patients and divided in both age groups, < 60 years or ≥ 60 years. Multiple logistic regression analysis showing odds ratio 95% CI and p value. Statistical significance was assumed at *p* ≤ 0.05.

				Death (all RSV infected patients)			Oxygen (all RSV infected patients)		
	All RSV infected patients^a^ *n*, (%)	< 60 years *n*, (%)	≥ 60 years *n*, (%)	Odds ratio	95% CI	*p* value	odds ratio	95% CI	*p* value
Immunosuppressed	114 (51.82)	61 (53.51)	53 (46.49)	1.24	0.52– 2.97	0.633	not tested		
Non‐immunosuppressed	105 (47.73)	31 (29.52)	74 (70.48)	0.81	0.34– 1.94	0.633	not tested		
Comorbidities RSV patients									
Diabetes	50 (22.73)	12 (24.00)	38 (76.00)	0.95	0.3–3	0.928	0.72	0.33–1.57	0.405
CHD	47 (21.36)	9 (19.15)	38 (80.85)	0.99	0.29–3.32	0.985	1.14	0.5–2.62	0.759
Hypertension	103 (46.82)	24 (23.30)	79 (76.70)	0.76	0.29–2	0.578	1.6	0.85–3.01	0.144
COPD	52 (23.64)	15 (28.85)	37 (71.15)	1.12	0.41– 3.06	0.827	2.92	1.43–5.95	0.003
Chronic cardiac insufficiency	36 (16.36)	5 (13.89)	31 (86.11)	1.19	0.31– 4.56	0.795	5.47	1.93–15.43	0.001

Abbreviations: CHD, chronic heart disease; COPD, chronic obstructive pulmonary disease.

^a^ For one patient, no information on immune status and for four patients no information on concomitant diseases was available.

In our RSV inpatient cohort, notably more patients aged 60 years or older required oxygen support (< 60 years: *n* = 16, 30.77%; ≥ 60 years: *n* = 36, 69.23%, *p* value 0.0047 Fisher's exact test) or died (< 60 years: *n* = 8, 28.57%; ≥ 60 years: *n* = 20, 71.43%, *p* value 0.68 Fisher's exact test) compared to the IAV/IBV‐infected patients (oxygen support: < 60 years, *n* = 98, 48.04%, ≥ 60 years: *n* = 106, 51.96%; death: < 60 years, *n* = 44, 44.90%, ≥ 60 years: *n* = 54, 55.10%) (Figure 3C,D and Supporting Information S1: Table 2). With 63.24%, slightly more RSV‐infected patients ≥ 60 years were admitted to the ICU than in the IAV/IBV group (52.25%), but this was not statistically significant (*p* value 0.126, Fisher's exact test). Interestingly, in RSV infection both male and female patients contributed to the increased percentage of hospitalized patients in the group of the over 60‐year‐old patients, but the highest percentages were seen in the group of RSV‐infected ≥ 60 year old men. In contrast, in the IAV/IBV cohort, gender distribution was nearly equal across both age groups (Figure 3C,D and Supporting Information S1: Table 2).

### 3.4 Hospitalized RSV‐Infected Patients Show a High Prevalence of Comorbidities

**Figure 4:**
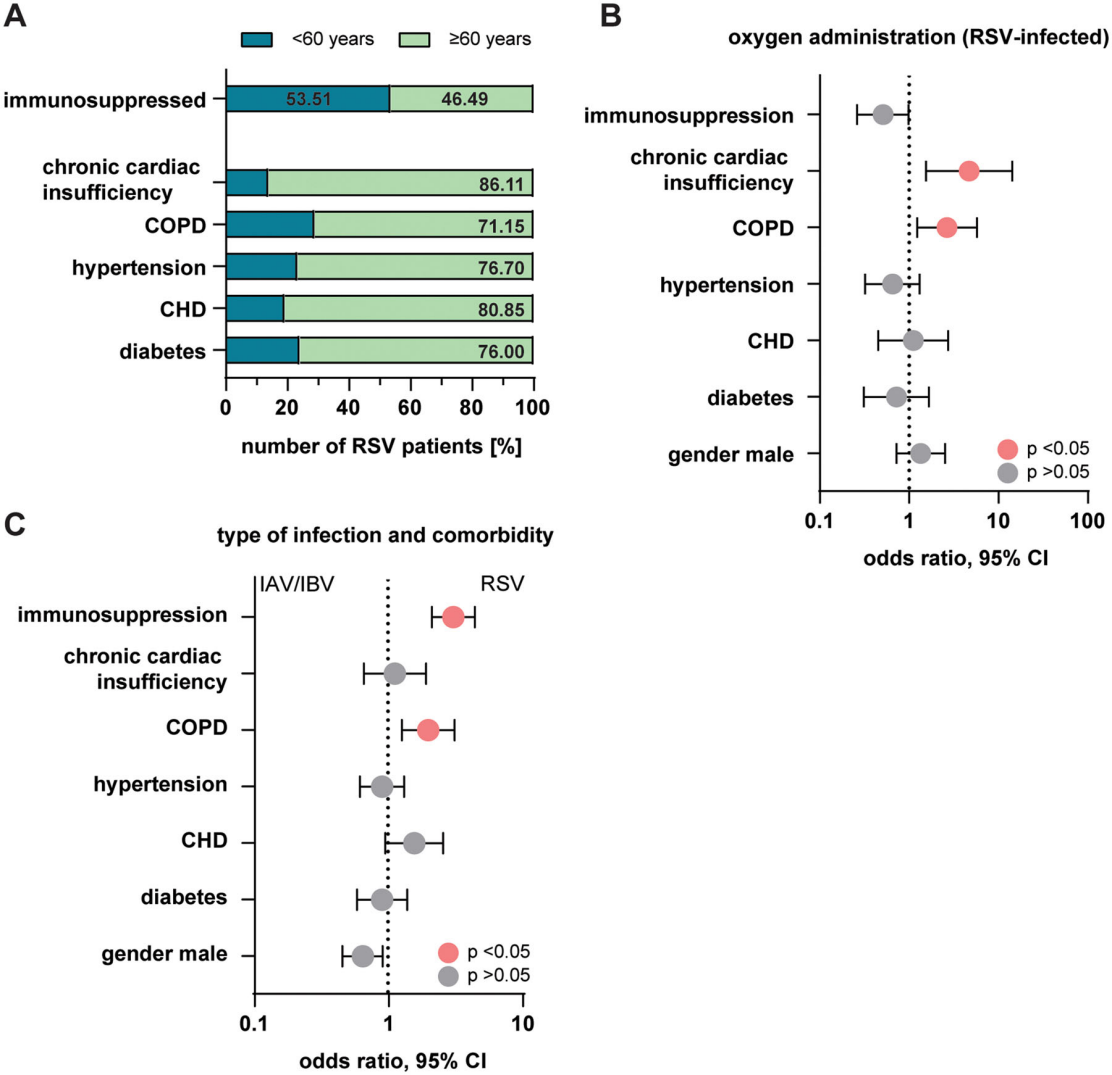
Comorbidities as possible risk factor of a severe course of RSV infection. (A) Frequency of different comorbidities present in the hospitalized RSV cohort. Shown are percentages for the age group ≥ 60 years (light green) and < 60 years dark green. For reasons of clarity, the percentage frequency is only shown for the age group ≥ 60 years. COPD, chronic obstructive pulmonary disease; CHD, chronic heart disease. (B) Multiple logistic regression analysis showing odds ratio and 95% CI as a forest plot for the probability of requiring oxygen due to a pre‐existing condition. Orange dots indicate statistical significance (*p* ≤ 0.05). (C) Multiple logistic regression analysis showing odds ratio and 95% CI as a forest plot for the association of a pre‐existing condition and infection type (RSV or IAV/IBV). Orange dots indicate statistical significance (*p* ≤ 0.05).

To address the influence of immunosuppression and comorbidities in our RSV‐infected cohort, we analyzed these parameters in more detail. Overall, 51.82% of the RSV‐infected patients were immunocompromised, of these slightly more patients were below the age of 60 (53.51%; ≥ 60 years 46.49%), (Figure 4A and Table 2). The most common comorbidity was hypertension with 46.82% of all RSV‐infected patients, with a notable higher number of patients over 60 years (76.7%) compared to patients < 60 years (23.30%) (Figure 4A and Table 2). Diabetes, chronic heart disease (CHD) and chronic obstructive pulmonary disease (COPD) were similarly distributed in their frequency (22.73%, 21.36% and 23.64%), with clearly more affected patients being over the age of 60. Chronic cardiac insufficiency was the least common comorbidity in RSV‐infected patients at 16.36% (Figure 4A and Table 2). As expected, COPD and chronic cardiac insufficiency were clear indicators for a higher probability of patients requiring oxygen after RSV infection (Figure 4B). However, in our RSV‐infected patient cohort none of these comorbidities showed a significant probability for an increased fatal outcome (Table 2).

Not unexpected, RSV‐infected patients who needed oxygen supply had the highest risk for a fatal outcome (odds ratio 14.98, 95% CI 3.33–67.47, *p* value < 0.001) (Supporting Information S1: Table 3). Likewise, immunosuppression (odds ratio 2.09, 95% CI 0.91–5.41, *p* value 0.128) and patients above the age of 60 years (odds ratio 1.4, 95% CI 0.53–3.73, *p* value 0.5) had a slightly increased risk for a fatal outcome, however these observations were not significant (Supporting Information S1: Table 3). Interestingly, male gender was not a risk factor in regard to a severe outcome (odds ratio 0.8, 95% CI 0.32–1.98, *p* value 0.626) (Supporting Information S1: Table 3).

To better assess the disease burden of RSV infection relative to IAV/IBV infection, we performed a multiple logistic regression analysis examining comorbidities in both hospitalized patient groups. The results showed that pre‐existing COPD (odds ratio 1.97, 95% CI 1.25–3.09 1.98, *p* value 0.003) and immunosuppression (odds ratio 3.02, 95% CI 2.09–4.37, *p* value < 0.001) were significantly more frequently associated with RSV‐infected hospitalization than with IAV/IBV (Figure 4C and Supporting Information S1: Table 4). No significant differences were found for chronic cardiac insufficiency, hypertension, CHD, diabetes or male gender (Figure 4C and Supporting Information S1: Table 4).

## 4 Discussion

The substantial burden of disease and high incidence of severe illness in young children with respiratory syncytial virus (RSV) infection is well known and widely studied, but remains understudied in adults. In recent years, awareness of RSV infection in older people has increased, partly due to changes in diagnostic testing strategies introduced during the COVID‐19 pandemic, which enabled more and earlier detection of RSV and identified RSV as source for severe disease progression in the elderly population. Previously, limited testing strategies often led to a late attempt to diagnose RSV, making virus detection difficult due to the short period of high viral load in affected elderly patients [20]. Consequently, severe disease courses could often not be linked to a specific pathogen leading to an underestimation of RSV‐positive cases and disease burden. Of note, the standard of care does not differ between different respiratory infections such as RSV or influenza virus and merely remains supportive, including fluids, antipyretics, and oxygen support when needed. With one exception, antiviral treatment for influenza virus, such as neuraminidase inhibitors or the cap‐dependent endonuclease inhibitor (baloxavir), can be administered to influenza virus positive patients, if the infection was diagnosed early in the course of disease. In contrast, no specific antiviral treatment is currently available for RSV infection.

In our retrospective observational study, we observed a sharp increase in positive RSV cases in the 2022/2023 season, which may be attributed to post‐pandemic impaired immunity but also to enhanced diagnostic testing. This was made possible by the introduction of point‐of‐care multiplex PCR tests in the ICU detecting the major respiratory viruses SARS‐CoV‐2, IAV, IBV and RSV during the COVID‐19 pandemic. Interestingly, a nationwide seasonal shift for RSV and influenza was observed after the second pandemic lockdown in Germany in the 2020/2021 winter. This shift was particularly evident in 2021/2022, when RSV infections increased already in calendar week 39 much earlier than in previous years, which typically showed the start of the season around weeks 51/52 [21]. One possible explanation for both the earlier onset and the overall rise in respiratory infections is the concept of “untrained immunity,” which may have resulted from widespread protective measures during the preceding pandemic winters [22]. In 2018, the number of IAV/IBV cases was notably higher compared to 2019 and 2020, largely due to the severe IBV season and a vaccine mismatch in 2017/2018 [23, 24]. In our cohort, we observed numbers consistent with nationwide data, including the high number of IAV/IBV cases in 2018 and the earlier onset of RSV and influenza seasons in 2021/2022, reinforcing the relevance of our cohort for this analysis (Figure 1C).

We found a similar mortality rate for RSV and influenza virus infections, albeit slightly higher numbers among Influenza A/B‐infected individuals (Figure 2). In addition, we demonstrated that RSV‐infected patients over the age of 60 had a particularly high risk of requiring oxygen supplementation, which increased the likelihood of a fatal outcome (Figures 3 and 4). Again, this is comparable to the disease burden of influenza virus infections, causing worldwide 3 to 5 million severe cases per year with an especially high mortality rate in people aged > 70 years (16.4 per 100,000 people) [25, 26, 27]. In our cohort, we found a similar high hospital mortality rate of 12.33% in RSV‐infected and 14.95% in Influenza A/B‐infected patients (Figure 2). The estimated mortality rate for influenza virus infections in the WHO European Region is 12%, with approximately 84% of these deaths occurring in the elderly population ( > 65 years) [28, 29]. These observations are in line with recent studies that also demonstrated a high disease burden of RSV infection in older populations comparable to that of influenza virus infections [4, 6, 30]. Here, very similar hospitalization rates and mortality was found. This suggests that RSV disease in adults causes a significant burden on healthcare systems, particularly in low‐ and middle‐income countries where access to medical care may be limited.

Immunosuppression and comorbidities are known to impact disease severity and progression in respiratory viral infections [31, 32]. In addition, age and the aging immune system is a well‐established risk factor for a poor outcome in infections with respiratory viruses [33, 34]. The University Medicine Essen serves as a tertiary care hospital with a specialization in transplantation, resulting in a patient population with a substantial proportion of transplanted and immunosuppressed individuals. Surprisingly, immune status alone did not appear to play a dominant role in influencing disease progression within our RSV‐infected cohort. Instead, age (≥ 60 years), COPD, and chronic cardiac disease emerged as the primary factors affecting requirements for oxygen supply and increasing the risk of a fatal outcome (Table 2 and Supporting Information S1: Table 3). Our cohort exhibited a much higher prevalence of underlying conditions such as COPD and CHD compared to the general population, underscoring their potential role in exacerbating the severity of RSV infections. The prevalence of COPD is estimated at around 10% worldwide and in Germany the estimated prevalence of cardiac vascular disease is about 12% [35, 36]. Moreover, the frequency of comorbidities increases with advancing age, highlighting a relationship between age and underlying conditions in shaping the clinical course of RSV infection. Previous studies also established COPD and cardiovascular disease as significant risk factors for severe RSV infections [4, 37, 38]. Similarly, patients who are severely immunosuppressed, such as those who have undergone solid organ or hematopoietic stem cell transplantation, were at elevated risk of requiring oxygen supply and intensive care upon RSV infection [39]. Additionally, increased rates of RSV‐related hospitalization and severe disease progression have been associated with other comorbidities, including chronic heart failure, cardiopulmonary diseases, and kidney disorders [40, 41]. Our analysis showed that COPD and immunosuppression were more frequently associated with hospitalized RSV‐infected patients than with IAV/IBV‐infected (Figure 4C). No significant differences were observed between the two groups with respect to other comorbidities. This observation highlights the comparable clinical impact of RSV and influenza viruses and underscores the importance of recognizing RSV as a pathogen of high clinical relevance in older populations.

In addition, immunosenescence is an aggravating risk factor in infectious diseases. Older individuals and people with comorbidities are at higher risk of severe infections and RSV infection is the third most commonly identified viral cause of hospitalization. Since there is no specific anti‐RSV treatment available the only option to tackle RSV infection are preventive measures. To date, two monoclonal antibodies, Palivizumab and Nirsevimab, are available for preventing RSV infection in infants but not the elderly. Recently effective active immunization has been approved and is now available and recommended for adults. However, active immunization remains challenging, particularly for immunocompromised individuals. In such cases, passive immunization with antibodies may offer a promising alternative for protecting this vulnerable population. Nevertheless, the new active vaccinations are an opportunity to protect elderly from serious illness and to reduce the burden on the healthcare system. Most likely, a broad implementation of RSV vaccines could lead to significant cost savings by preventing severe illness and reducing hospitalizations. In addition, comprehensive cost‐effectiveness analyses are crucial and should be a focus of future research to ensure informed decision‐making and policy development, especially for vaccine introduction in low‐income countries. But so far, due to the lack of real‐world data, vaccine recommendation in Germany refers to people over the age of 75 or at the age of 60 years with underlying comorbidities. The Spanish NeumoExperts Prevention Group advised vaccination for individuals aged 60 and older, as well as for healthcare workers who face a higher risk of acquiring and transmitting RSV [42]. In general, especially vulnerable patients with underlying comorbidities and the elderly should be prioritized for vaccination.

Overall, a limitation of our study is the relatively smaller number of RSV‐infected patients in this cohort compared to those infected with IAV/IBV. This discrepancy may result in a bias in our analysis, as relative differences may not accurately reflect the overall disease burden in RSV and IAV/IBV infection, due to the imbalance in patient numbers. Furthermore, our findings may be influenced by a selection bias, as patients at a university hospital have frequent comorbidities and present more often to the emergency department or outpatient clinics, while individuals with only mild respiratory symptoms are typically underrepresented. Given the hospital's role as a referral center, a higher proportion of patients presenting with severe disease is expected, which may limit the generalizability of our findings to the broader population. However, within high‐risk groups, these results provide valuable insights into RSV disease burden and healthcare impact. Notably, the proportion of patients experiencing significant RSV disease burden is substantial and comparable to those with complications upon IAV/IBV infections. As this is a retrospective observational study, some degree of missing data is inevitable, which may influence the robustness of our statistical analyses. For example, a comprehensive analysis of a standardized severity scoring system for vital parameters, such as SOFA (sequential organ failure assessment) was not possible as these data were rarely documented in the medical records. Further prospective studies are needed to validate our findings. Nonetheless, our retrospective analysis highlights RSV as a serious condition in adults, with a high disease burden and mortality rates, particularly among older individuals. The data demonstrate this for individuals aged 60 and older, suggesting that current vaccine recommendations focusing on those over 75 may be too restrictive. Consequently, our data supports the hypothesis that individuals over 60 years of age independent of comorbidities including immunosuppression, should be prioritized as a key target group for RSV vaccination recommendations.

## Author Contributions

**Georgii Trifonov:** data collection, writing – review and editing. **Erik Büscher:** data collection, writing – review and editing. **David Fistera:** data collection, writing – review and editing. **Clemens Kill:** data collection, writing – review and editing. **Joachim Risse:** data collection, writing – review and editing. **Christian Taube:** resources, writing – review and editing. **Daniel Todt:** methodology, software, visualization, writing – review and editing. **Ulf Dittmer:** conceptualization, methodology, resources, writing – original draft, writing – review and editing. **Carina Elsner:** conceptualization, methodology, investigation, formal analysis, visualization, writing – original draft, writing–review and editing.

## Conflicts of Interest

The authors declare no conflicts of interest.

## Supporting information

Revision RSV Supplemental material.

## Data Availability

The data that support the findings of this study are available on request from the corresponding author. The data are not publicly available due to privacy or ethical restrictions.
