# Supplementary material for: Disease Burden of RSV Infection in Adult Patients in Comparison to Influenza Virus Infection
Source: J Med Virol. 2025 Apr 30;97(5):e70373. doi: 10.1002/jmv.70373 (PMC12041907; doi:10.1002/jmv.70373)
Supplement: Supplementary file 1 — Revision RSV Supplemental material. [file JMV-97-e70373-s001.pdf]

Supplementary material to

Disease burden of RSV infection in adult patients in comparison to Influenza virus infection

Georgii Trifonov[1], Erik Büscher[2], David Fistera[3], Clemens Kill[3], Joachim Risse[3], Christian Taube[2], Daniel Todt[4,5], Ulf Dittmer[1,*], Carina Elsner[1,*]

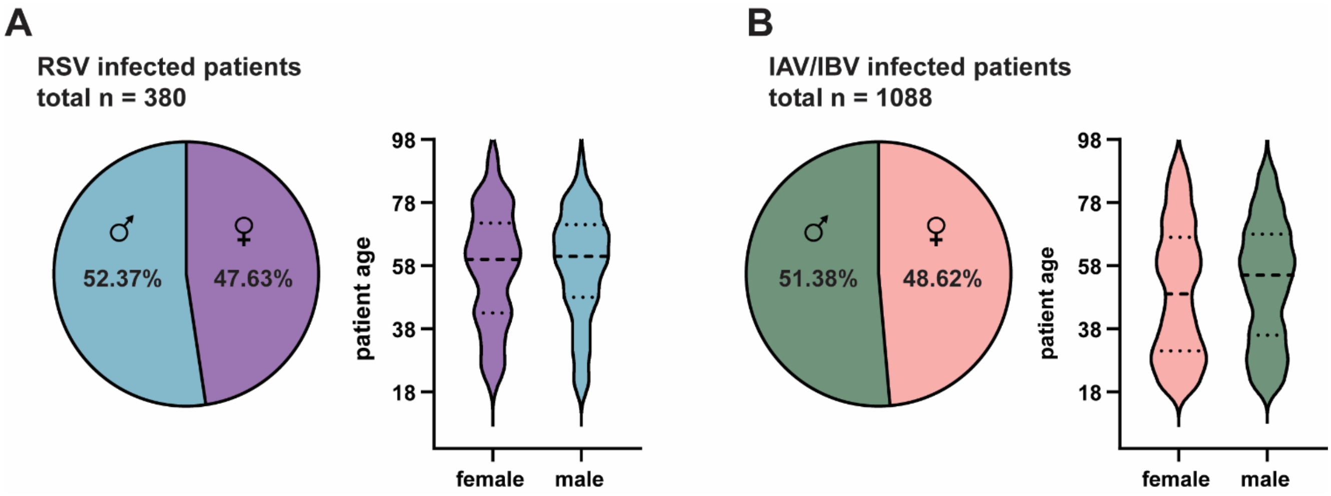

Supplemental Figure 1: Frequency of gender distribution and corresponding age distribution of both in- and outpatients. (A) RSV-infected patients. (B) IAV/IBV-infected patients.

Supplemental Table 1: Characteristics of inpatients. Summarized are the distribution of hospitalized patients and gender, as well as necessity of oxygen supply and the disease outcomes for the two cohorts with RSV infection and IAV/IBV infection. Further the admission rate to intensive care unit (ICU) as well as the number of patients that needed invasive ventilation (IV) are given. The term “oxygen supply without IV” includes non-invasive ventilation, high flow oxygen admission and nasal cannula/oxygen mask. The p value was calculated for RSV compared to IAV/IBV using Fischer’s exact test, statistical significance was assumed at p <0.05.

**Characteristics of inpatients**	**RSV n (%)**	**IAV / IBV n (%)**	**p value**
all cases	380 (25.90)	1088 (74.10)	
inpatients	227 (59.74)	649 (59.65)	n.s
outpatients	153 (40.26)	439 (40.35)	
**gender distribution**			
male	111 (48.90)	347 (53.47)	n.s.
female	116 (51.10)	302 (46.53)	n.s.
**oxygen administration**			
oxygen supply	52 (22.91)	204 (31.43)	0.017
no oxygen supply	175 (77.09)	445 (68.57)	
**outcome**			
death	28 (12.33)	98 (15.10)	n.s.
regularly discharged	163 (71.81)	436 (67.18)	
transfer to another hospital / rehabilitation	28 (12.33)	97 (14.95)	
against medical advice	7 (3.08)	13 (2.00)	
other reasons	1 (0.44)	5 (0.77)	
**ICU admission**			
	68 (30.91)	222 (36.04)	n.s.
	152 (69.09)	394 (63.96)	
**proportion of IV oxygen supply during ICU stay**			
IV	35 (51.47)	151 (68.02)	0.0214

Supplemental Table 2: Characteristics of inpatients in the age groups <60 and ≥60 years. Summarized are the distribution of hospitalized patients and gender as well as the necessity of oxygen supply, outcome and the admission rate to the intensive care unit (ICU) for the two cohorts with RSV infection and IAV/IBV infection. The p value was calculated for RSV (≥60 years) compared to IAV/IBV (≥60 years) using Fischer's exact test, statistical significance was assumed at p <0.05.

	**<60 years**		**≥60 years**		
	**RSV**	**IAV/IBV**	**RSV**	**IAV/IBV**	**p value**
inpatients, n (%)	94 (41.41)	333 (51.31)	133 (58.59)	316 (48.69)	0.01
outpatients, n (%)	84 (54.90)	340 (77.45)	69 (45.10)	99 (22.55)	
**INPATIENTS**					
**gender distribution**					
male, n (%)	42 (37.84)	174 (50.14)	69 (62.16)	173 (49.86)	
female, n (%)	52 (44.83)	159 (52.65)	64 (55.17)	143 (47.35)	
**oxygen administration per age group**					
oxygen supply, n (%)	16 (30.77)	98 (48.04)	36 (69.23)	106 (51.96)	0.0047
**outcome per age group**					
death, n (%)	8 (28.57)	44 (44.90)	20 (71.43)	54 (55.10)	n.s.
**ICU admission rate per age group**					
ICU admission, n (%)	25 (36.76)	106 (47.75)	43 (63.24)	116 (52.25)	n.s

Supplemental Table 3: Multiple logistic regression analysis to investigate the influence of male sex, immunosuppression, need for oxygen supply or age ≥60 years on the variable outcome death, to predict the probability of being dead in the context of RSV infection. The model as a whole was significant, chi-square 22.81, $p < 0.001$, $n = 220$. Shown are the coefficient B, standard error, z value, p value, odds ratio and 95% CI. Statistical significance was assumed at $p < 0.05$.

	coefficient B	standard error	z value	p value	odds ratio	95% CI
constant	-4.56	0.88	5.17	<0.001	0.01	0.0 – 06
gender male	-0.23	0.47	0.49	0.63	0.8	0.32 - 1.98
immunosuppressed	0.74	0.49	1.52	0.13	2.09	0.81 – 5.41
oxygen supply	2.71	0.77	3.52	<0.001	14.98	3.33 – 67.47
age category ≥60 years	0.34	0.50	0.67	0.5	1.40	0.53 – 3.73

Supplemental Table 4: Multiple logistic regression analysis to investigate the influence of male sex, immunosuppression, chronic cardiac insufficiency, COPD, hypertension, CHD and diabetes to predict the probability of being hospitalized due to RSV or influenza virus infection. The model as a whole was significant, chi-square 118.19, $p < 0.001$, n = 797. Shown are the coefficient B, standard error, z value, p value, odds ratio and 95% CI. Statistical significance was assumed at $p < 0.05$.

	coefficient B	standard error	z value	p value	odds ratio	95% CI
constant	-1.97	0.35	5.7	<0.001	0.14	0.07 – 0.27
gender male	-0.45	0.18	2.57	0.01	0.64	0.45 – 0.9
diabetes	-0.11	0.22	0.52	0.6	0.89	0.58 – 1.37
CHD	0.44	0.25	1.72	0.08	1.55	0.94 – 2.54
hypertension	-0.12	0.19	0.62	0.54	0.89	0.61 – 1.3
chronic cardiac insufficiency	0.1	0.27	0.37	0.71	1.11	0.65 – 1.89
COPD	0.68	0.23	2.94	0.003	1.97	1.25 – 3.09
immunosuppression	1.1	0.19	5.87	<0.0010	3.02	2.09 – 4.37
